# Impact of Social Exclusion on Customer Participation in Innovation: Role of Customer-Company Identification

**DOI:** 10.3389/fpsyg.2021.747924

**Published:** 2021-11-12

**Authors:** Zhang Hui, Mou Yupeng, Zhang Chenglong, Li Haiqin, Guo Daomeng

**Affiliations:** ^1^School of Economics and Management, Hubei Engineering University, Xiaogan, China; ^2^School of Management, China University of Mining and Technology, Xuzhou, China

**Keywords:** customer participation, social exclusion, innovation, customer-company identification, mediating effect

## Abstract

In a social context, customer participation in the innovation process is often accompanied by social exclusion situations, which are generally believed to have a negative impact on individuals. However, research results and marketing practices show that social exclusion can also exert a positive influence on creativity, product selection, perceived risk, and so on. Through two experimental studies, this research explores the relationship between social exclusion and customer participation in innovation. It finds that social exclusion has a positive influence on customer participation in innovation and that customer-company identification mediates this relationship.

## Introduction

With the rise of open innovation, customer participation in innovation has evolved from individual behaviors to social ones involving interactive communication (Khan et al., 2019; Hofstetter et al., 2021). Meanwhile, in recent years, the innovation of consumer electronics products and daily use products have often been associated with socially excluded individuals who have little contact with the real society and shut themselves off in their own worlds (Su et al., 2017). In our daily life, we often feel the scenes of social exclusion. No birthday invitation from friends, no thumb up circle of friends, and these scenes may make us feel excluded. These individuals may be actively engaged in their areas of interest through online networks and other channels, and they may generate a substantial number of ideas (Kim et al., 2013). Then, question arise does social exclusion, affect customers’ participation in innovation?

Social exclusion refers to the state of being marginalized and isolated (Twenge et al., 2001) it deprives individuals of their sense of belonging and reduces their sense of control (Bhalla and Lapeyre, 1997). To rebuild social connections, individuals alter their cognitive responses and preferences through social information processing (Popay et al., 2010; MacDonald and Macdonald, 2020). It is generally believed that social exclusion can engender two kinds of consumer behavior reactions (Vinuales and Thomas, 2021). Positive consumer reactions include prosocial behavior and donations, while negative consumer reactions can include declining evaluations and aggressive behavior, among others. Social exclusion is the state of an individual being deprived of or absent from social activities (Dorsner, 2004); participation in social activities is thus regarded as an important criterion for exclusion (Hills et al., 2002). The marketing literature supports the idea that participation in social activities increases consumers’ affinity behavior in brand community participation (Muniz and O’guinn, 2001), but some studies have also mentioned the relationship between participation in social activities and customer participation in innovation (Auh et al., 2019). Overall, the existing literature pays little attention to the impact of social exclusion on customer participation. However, in practice, social exclusion can affect various customer behaviors; academically, the mechanism of social exclusion affecting customer participation is still unclear, and the study of customer behavior from social factors is still lacking.

There are two advantages to emphasizing the influence of social factors (e.g., social exclusion) in the process of customer participation in innovation. The first is that it allows for a better understanding of the reasons behind this kind of customer participation (Wang and Yu, 2019). In the context of open innovation and mass customization, customer participation is based on social interaction behavior. If we focus only on the economic and psychological aspects of the individual and ignore the individuals’ associations in society, the result will be a lack of research externality (Perry-Smith and Shalley, 2003; Kakar and Khan, 2020; Khan et al., 2020a). The second advantage is facilitating an enhanced understanding of the source of participation in innovation (Ali et al., 2020). The research on this subject has been conducted from two perspectives: the customer perspective and the market one. The customer perspective considers customer knowledge, input, and other factors, while the market perspective considers the elimination and selection mechanisms of innovative ideas, the cardinality principle of the quantity of creativity, and so forth (Mustak, 2019). Since the essence of innovation is to restructure the relationships between different things, it may be more appropriate to study activities and behaviors from a social perspective (Williams and Hubbard, 2001; Johansson, 2004; Xiongfei et al., 2019). At the same time, because of customers participation in enterprise activities and the need to connect with the enterprise (Khalifa and Shukla, 2017), we believe that customer-company identification will play a role in the intermediate mechanism.

The purpose of this study is to explore the influence of social exclusion situations on customer participation in innovation and the intermediary role of customer-company identification. The remainder of the paper is divided into three parts. The first part contains the literature review and hypotheses. The literature on social exclusion, customer-company identity, and customer participation is reviewed and the relevant research hypotheses are proposed. The second part of the paper presents the experimental study, which is used to test the hypotheses. The third part discusses the theoretical and practical value of the article, its shortcomings, and future research directions.

## Literature Review

### Social Exclusion

In daily life, social exclusion is a common phenomenon which is defined as people’s affiliation needs are hindered, they may experience a state of deprivation (Popay et al., 2010). Stress and anxiety tend to increase, precipitating a psychological experience akin to physical pain (Cao et al., 2018). People may even begin to feel that others do not see them as human beings (Bastian and Haslam, 2010; Khan et al., 2020a), or they may lose the sense that life has meaning (Twenge et al., 2003; Khan, 2021).

Currently, there are no generally accepted conclusions about social exclusion behaviors (Gardner et al., 2005; Maner et al., 2007; Williams, 2007). Broadly speaking, however, two kinds of behaviors appear to exist. First, social exclusion seems to increase prosocial behavior. People who are socially excluded show greater interest in meeting new friends through social services and are more willing to work with others (Maner et al., 2007). They unconsciously imitate others (Lakin et al., 2008), are more concerned about social events (Pickett et al., 2004; Khan et al., 2020b), focus on improving their social skills, and use less stereotypical judgment (Claypool and Bernstein, 2014). In the field of consumer behavior, social exclusion has been found to increase the cost of enhancing affinity (Mead et al., 2011). It boosts individuals’ preferences for nostalgic products that accentuate individuals’ relationship with their past (Loveland et al., 2010), promotes active engagement in volunteer work, and inspires donations of money or blood (Weinstein and Ryan, 2010). Second, social exclusion can also increase aggressive and antisocial behavior (Twenge et al., 2007; Khan et al., 2020b). Individuals who have been excluded from society tend to negatively evaluate the work of people who offend them (Twenge and Campbell, 2003; Khan et al., 2019), and they increase conspicuous consumption to attract attention (Rucker and Galinsky, 2009). Food-based studies have found that people who were socially excluded would give relatively unappealing snacks to their interactive partners (Chow et al., 2008) and distribute more chili sauce to people expressing a dislike for spicy food (Warburton et al., 2006). For enterprises, the positive and negative effects caused by social exclusion are more because social exclusion is a complex psychological state, and different behaviors will occur due to the gap between scenarios (Lee and Shrum, 2012; Khan et al., 2021).

Presently, researchers are paying considerable attention to the influence of social exclusion on consumer product selection and preferences. However, from the perspective of market reality, service logic will gradually occupy the dominant position in the market, and understanding social exclusion’s influence on customer participation can help to extend the research conclusions in the service field. Through a mediating effect, social exclusion helps to clarify the mechanism of customer participation in innovation.

### Customers’ Willingness to Participate in Innovation

Innovation is a key process that affects the survival and success of an enterprise or organization; it is an important source of competitive advantage (Brown and Eisenhardt, 1995). With continuous advances in technology, products have improved and become more sophisticated. However, it may be challenging for enterprises to fully understand the diversified needs of customers and to gauge whether the market will accept new products. Thus, innovation is an activity that carries a high risk for an enterprise. To carry out successful innovation and enhance the value of products, an enterprise must recognize the importance of customer participation in innovation.

Why involve customers in innovation? Currently in the literature, there are three widely accepted views on customer participation in innovation (Rose et al., 2021). First, ideas about customer participation are derived from the service field (Prior et al., 2019; Vesal et al., 2021). In the creation and delivery of services, customer interaction and service invisibility are important features, and there is a fairly large class of services that depend on customer participation. The second view is concerned with the material benefits of customer participation, such as price discounts or functional agreements. The third view focuses on participation’s psychological benefits, such as that it promotes a customer’s sense of control, enhances his or her sense of identity, or strengthens his or her self-esteem. The innovation process of this paper focuses more on the social factors in the participation process, namely, the innovation process provides the interaction between customers and the connection with the enterprise. Customer participation can bring more creativity to enterprise innovation, but from a professional perspective, customers still lack a deep understanding. Therefore, the form that enterprises prefer customers to participate is by letting customers choose themselves and clearly understand customers’ needs. In these processes, customers can also communicate with other customers or enterprises by participating and using their self-choices.

Social exclusion, on the other hand, can erode self-esteem and detract from one’s sense of belonging, control, and identity (Zadro et al., 2004), threatening relationship demands and control needs. To increase their satisfaction with the social environment, individuals who are socially excluded will actively seek out new relationships and rebuild their sense of belonging (Baumeister and Leary, 1995). The objects of the reconstruction attribution are not necessarily individuals in society but may also be non-social entities, such as furniture, trees, or other objects (Epley et al., 2008). In other words, individuals experiencing social exclusion will be highly motivated to cultivate social connections even with alternative non-human sources. Ultimately, individuals will be more sensitive to information that can provide social connections. People will use different strategies to cope with social exclusion (Williams, 2007), including increasing social receptivity, reinvesting in social connections (Maner et al., 2007), enhancing attentiveness to and memory of social information, and automatically attending to customer-company identity information (DeWall et al., 2011).

There is an abundance of research on customers and enterprises and their reasons for participating in innovation (Chang and Taylor, 2016). However, many areas of research require further attention, considering rapidly changing market practices. Primarily, there is a lack of research on social factors. Studies on the antecedents of customer participation in innovation suggest that customers are driven by economic and psychological benefits. However, open innovation (e.g., crowdsourcing and mass customization) implies that innovation participation should not be the only consideration in the study of interactions between customers and enterprises (Camacho et al., 2019; Simpson et al., 2021). Rather, these interactions also depend on important social factors (Ashton-James and Chartrand, 2009). To understand the role of social exclusion as an antecedent, we focus on effects from relationship changes between customers and other customers and between customers and enterprises. This approach contributes to a more comprehensive understanding of the mechanism behind customer participation in innovation. Another issue that warrants further investigation is the impact of negative factors on customer participation (Zhang et al., 2020). More scholarly attention has been paid to positive factors, but from a consumer behavior perspective, all causes of behavior are not necessarily positive, and in certain cases, negative factors may actually have a greater impact on behavior. Thus, this research on the effects of social exclusion on customer participation, and it sheds light on the causes of consumer behavior.

On the surface, customer participation in innovation seems like a type of customer or enterprise behavior, but in actuality, open innovation, mass customization, and other forms of customer participation are more likely to be social behaviors (Hills et al., 2002). Customer participation brings enterprises into closer contact with people and societies (Dorsner, 2004). Meanwhile, social exclusion detracts from individuals’ sense of belonging, which originates from self-categorization, and the choice to participate in innovation is itself a kind of classification process. For example, participation activities offered by strong brands, such as Apple and Intel, provide customers with a new means of self-classification, and this can enhance their sense of belonging. Customer participation also enables enterprises to transfer the power of innovation to their customers. Through their participation, customers can serve as sources of originality and creativity, choose product prototypes, contribute knowledge, and achieve a degree of control over the innovation process.

Therefore, we believe that a positive relationship exists between social exclusion and customer participation in innovation, and the following hypothesis is proposed.

H1: *Customers will be more willing to participate in innovation in the context of social exclusion than in non-social-exclusion situations*.

### Customer-Company Identification

Customer-company identification refers to the degree of connection that customers perceive with an enterprise (Haumann et al., 2014). It can also refer to the customer’s perception that the enterprise’s identity is congruent with his or her own identity in terms of self-referential positioning (Einwiller et al., 2006). Individuals who identify with a certain group strive to become a part of that group by imitating other members, adjusting their own personality accordingly (Sanford, 1955). During the establishment of customer and enterprise identity, a customer’s and an enterprise’s identity become connected and intertwined (Bagozzi et al., 2012). This process enables a customer to use an enterprise identity to shape his or her social identity to meet social self-definition needs. When the identity characteristics of an enterprise can satisfy a customer’s needs for self-definition, the customer will be attracted to the enterprise and integrate the identity characteristics of the enterprise into his or her social identity (Bagozzi and Dholakia, 2006). Furthermore, when customers identify with an enterprise, they will interpret enterprise behavior more positively, which reinforces their commitment and positive attitude toward the enterprise (Brown et al., 1993). This process encourages loyalty behavior and repeat purchases. At the same time, customer-company identification gives customers some immunity to negative information about the enterprise (Einwiller et al., 2006).

A sense of identity is closely connected with a sense of belonging and strongly contributes to the sense of being a part of a group (Kazançoğlu and Dirsehan, 2014). Individuals can cultivate and express their social identity by strengthening their associations with enterprises or by forming membership relationships in the classification of social entities (Ashforth and Mael, 1989). Social exclusion contributes to these processes in that it results in a lack of belonging and a threat to individual relational demands, thereby fostering an urgent need for customer-company identification. In terms of the antecedents of customer-company identification, past studies have focused on factors, such as the attraction of the enterprise or the agreement between an enterprise and a customer (Kang et al., 2014). However, from the point of view of customers, customer-company identification is the process of a customer seeking to meet his or her need for self-ascription (Ahearne et al., 2005). The establishment of customer-company identification is based on a customer’s selective and volitional initiative (Bhattacharya and Sen, 2003). Customers who are socially excluded are better able to resist the adverse effects of social exclusion when they choose specific brands. On the one hand, excluded consumers compensate for social belonging by choosing specific brands to increase opportunities for social recognition (Lee and Shrum, 2012). Moreover, excluded consumers tend to consider an enterprise as a relationship object, and they compensate for a lack of belonging through interaction with the enterprise brand. Social exclusion leads people to become more sensitive to social clues and more motivated to seek social acceptance; socially excluded individuals seek out new friendships to restore their sense of social connection (DeWall et al., 2009a). For individuals who are socially excluded, an enterprise’s invitation to participate in innovation is seen as a positive and friendly gesture, and the individuals may perceive that the possibility of reconstructing a sense of belonging will be greater if they participate. In this sense, customer-company identification gives individuals a sense of worth or value, and in exchange for this value, the customer is more likely to affirm the behavior and overall concept of the enterprise. At the same time, customers become more willing to join forces with the enterprise with it, thereby improving their self-esteem (Dommer et al., 2013).

Therefore, we believe a positive relationship exists between social exclusion and customer-company identification, and the following hypotheses are proposed.

H2: *In a customer participation environment, a customer in a social exclusion situation will perceive higher customer-company identification than a customer in a non-social-exclusion situation*.

Customer-company identification is based on a close relationship between a customer and the enterprise. Through this connection, a customer derives a sense of belonging and improves his or her self-worth. Customer participation is a kind of behavioral and psychological involvement, and the degree of correlation between enterprises and customers is high. Perhaps, customers even see the enterprise as an extension of themselves (Press and Arnould, 2011). Customers are then more willing to participate in the enterprise’s activities and plans for business. Customer participation further increases the interaction between customers and enterprises, and ultimately, they reach a consensus on the process of innovation—this is also a manifestation of the customer-company identification. In sum, customer-company identification embodies all aspects of the close relationship between a customer and an enterprise. Customers with a higher level of customer-company identification will be more willing to devote their energy to the innovation activities of the enterprise. Consequently, efficiency in communication processes during innovation will be higher, and customers will be willing to put forth more cognitive effort to generate new ideas (Wang and Yu, 2019).

Therefore, we believe that a positive relationship exists between customer-company identification and creativity, and the following hypotheses are proposed.

H3: *Relative to customers with a low level of customer-company identification, consumers with a high level of customer-company identification are more willing to participate in innovation*.

H4: *Customer-company identification mediates the H1 relationship*.

### Experimental Research

In this paper, two experiments are conducted to test the above hypotheses. These involve different manipulation methods, and the influence of other variables is by all means excluded. At the same time, in the process of the experimental design, customers participate in specific situations that approximate real situations as much as possible to obtain optimal validity.

### Research Study: 1

#### Research Design and Participants

This study explored customer participation in innovation situations, adopting a social exclusion (high and low) between-group design method to test the above-stated hypotheses. The randomized block design method was used to test the influence of customer-company identification on the relationship between social exclusion and a customer’s willingness to participate in innovation (Piepho, 1997). Direct notification was used to indicate the initiation of social exclusion perception (Press and Arnould, 2011), and the measurement of customer-company identification used the scale in mature literature (Einwiller et al., 2006).

In Hubei, through the campus network, the participants were recruited in the name of innovative discipline competition and each participant was given a small gift worth 10 Yuan after the experiment. A total of 127 college students between the ages of 17 and 27 participated in the study. After disqualified questionnaires were eliminated, the number of effective participants was 113, and the participants were randomly divided into groups. They were told that the school was responding to a request by the state for “Mass Innovation and Entrepreneurship” by conducting a survey of university students’ willingness to innovate and start businesses. The manipulation of social exclusion exploits the way of the mature literature (Nezlek et al., 1997). The students voluntarily formed innovative and entrepreneurial teams of more than three people to participate in a third-party company’s public service innovation activities and complete the relevant information forms. When the socially excluded participants returned the form, they were informed *via* written feedback that no one was willing to team up with them and they had to work alone. We informed the non-excluded participants that they had successfully formed a team. At the same time, the participants were asked to read relevant information on customer participation in the innovation. The enterprise participating in innovation was hypothesized to be the “Mai Qi Toy Company,” and the introduction was adapted from a real company profile. The innovation situation asked the participants to participate in the design of a toy for public welfare. The design material was classified according to the design process. Each material provided 3–5 alternative choices, and the participants were guided by professionals through the Internet, *via* telephone, and so on. Finally, the participants were asked to complete questionnaires that assessed social exclusion, customer-company identification, and customer willingness to participate in innovation, and they were told that the recruitment results would be mailed to them.

#### Control Test

Social exclusion used the form of self-reporting (Kim et al., 2013; e.g., “I felt that I was excluded from the group”). There were three questionnaire items that used a 5-point scoring system. The reliability coefficient of the scale was 0.81, and there was a significant difference between the two groups (Mhigh=3.47, Mlow=2.53; *t*=9.36, *p*<0.01). Customer knowledge was also tested, as factors, such as design knowledge, were involved in the innovation situation (Wynder, 2007). For this test, there were six items using a 5-point scoring system. The reliability coefficient of the scale was 0.84, and there was no significant difference between the two groups (Mhigh=3.96, Mlow=2.97; *t*=12.1, *p*=0.64). For the virtual “Mai Qi Toy Company,” all the participants were tested for familiarity, and 91.3% of the participants had not heard of the company.

#### Results

A maturity scale was used to measure customer-company identification and the customer’s willingness to participate in innovation, with reliabilities of 0.87 and 0.84, respectively (Bendapudi and Leone, 2003; Hunter and Garnefeld, 2008). With social exclusion as an independent variable, the analysis of variance was carried out with customer-company identification and customers’ willingness to participate in innovation. The results showed that social exclusion had a significant impact on customer-company identification (*F*(1,111)=9.42, *p*<0.05) and customer participation in innovation (*F*(1,111)=5.16, *p*<0.05). At the same time, customer-company identification was grouped *via* the mean 3.27 level, and data within one standard deviation were removed. *T*-value comparisons were made for the customers’ willingness to participate in the innovation (Mhigh=3.89, Mlow=3.25), and the results showed significant differences between the two groups *t*=12.42, *p*<0.01. The above effects were all significant, indicating that the social exclusion, customer-company identification, and customer participation in innovation interact with each other, providing the possibility for a test of an intermediary role.

**Figure 1:**
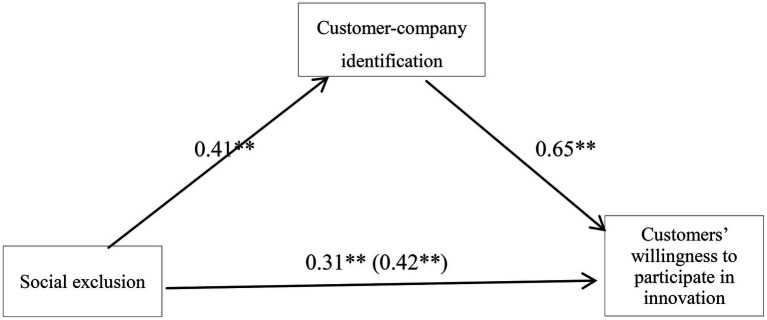
The intermediary model of customer-company identification and the regression coefficient of each path. ^**^p<0.01.

According to the intermediary variable test procedure, the social exclusion (SR) was taken as the independent variable, the customer-company identification (CE) was taken as the intervening variable, and the customers’ willingness to participate in innovation (CP) was taken as the dependent variable in the construction of the regression analysis. First, the customers’ willingness to participate in innovation was taken as the dependent variable and social exclusion was taken as the independent variable to construct the regression analysis, leading to the regression equation 1: CP=cSR+e_1_. Next, customer-company identification was taken as the dependent variable and social exclusion was taken as the independent variable to construct the regression analysis, leading to equation 2: CE=aSR+e_2_. Finally, the customers’ willingness to participate in innovation was taken as the dependent variable and social exclusion and customer-company identification were taken as the independent variables to construct the regression analysis, thereby giving rise to regression equation 3: CP=c’SR+bCE+e_3_. The normalized regression coefficient of each path of the model is shown in Figure 1. The results showed that social exclusion had a significant positive predictive effect on customers’ willingness to participate in innovation (*β*=0.42, *p*<0. 05). When customer-company identification was put into the regression equation, the regression coefficient of social exclusion on customers’ willingness to participate in innovation decreased despite still being significant (*β*=0.37, *p*<0.05), indicating that customer-company identification played a partial intermediary role of social exclusion in the prediction of the customers’ willingness to participate in innovation. The indirect action of the model was (0.41)×(0.65)=0.27. Customer-company identification and social exclusion were incorporated into the equation, and then, the obtained R^2^ was 0.59. Therefore, the intermediary role of customer-company identification in social exclusion and customer participation in innovation was significant.

The experimental results support the relevant assumptions. First, social exclusion positively affects customers’ willingness to participate in innovation. Social exclusion situation can reduce personal social belonging, and customer participation in innovation is a way to integrate more effectively into society. Therefore, excluded customers are relatively more willing to participate. However, the literature suggests that social exclusion produces two different tendencies (Twenge et al., 2001), the first one being that excluded individuals become more willing to integrate into society. In this experiment, the participants did not know each other, so the rejection was likely to be understood as a random event, and the above data results emerged. The other tendency is that personal differences with others become more pronounced, especially when the source of rejection is stable (Wan et al., 2014), and thus, the participation could cause the customers to be voluntarily isolated.

Second, the mean grouping method is adopted in the data analysis of the relationship between customer-company identification and the customers’ willingness to participate in innovation, increasing the significance of the results to a certain extent. However, this relationship was demonstrated in several other studies on customer participation, and the mediating effect analysis was not conducted this way. Thus, the conclusion of this paper is not affected.

Third, the role of customer-company identification is clarified. Different customers interpret the situation differently; therefore, the social exclusion affects customer-company identification. Customers who are socially excluded pay more attention to the clues regarding identity and belonging (Claypool and Bernstein, 2014). At the same time, the positive influence of customer-company identification on the willingness to participate can be interpreted to mean that identity factors are present in the antecedents of participation.

Fourth, regarding an intermediary role, customer-company identification partly mediates the impact of social exclusion on customer participation in innovation. On the one hand, social exclusion not only affects the identification of people with the enterprise, but it also affects belonging and self-cognition. Thus, there will be other effects (Dommer et al., 2013). On the other hand, the difficulty in obtaining a complete mediation result was due to problems in the data collection and sample.

### Research Study: 2

#### Research Design and Participants

Research 2 used an alternative experimental scenario. Social exclusion started with a networking situation to further test the hypotheses. On the one hand, customers often participate in innovation through a network. This is very convenient for both enterprises and customers, and thus, they use it more and more often. On the other hand, many social exclusion situations can arise in network communication, which are often observed in market practice (Williams and Hubbard, 2001). In addition, measuring willingness through self-reporting may lead to some differences between results and actions (Glasman and Albarracín, 2006). Therefore, research 2 changes the method of measurement, such that the input situations in the actual participation behaviors are employed in the measurement to exclude the self-reporting bias.

The experiment still uses the between-group design method of social exclusion (high and low), following the design of Wan et al. (2014). A total of 93 college students in Hubei were recruited through the campus network. These students were in the 18–25 age range. Disqualified questionnaires were eliminated, and the remaining number of effective participants was 89. All processes were completed in the computer room. The participants were asked to read online dating stories in first person on the computer, and then, they imaged that they themselves experienced the described situation. The main content was as follows: “I found three people who know a lot about digital products in the WeChat digital interest group. I made requests to be friends, and 2days later I received replies.” In the condition of social exclusion, the replies were that three people rejected the request; in the condition of non-social-exclusion, three people accepted the request. After reading the story, the participants were asked to describe in detail how they felt after experiencing this situation; this was to strengthen the manipulation of social exclusion (Rucker et al., 2011).

Subsequently, the participants were asked to complete a questionnaire about digital product innovation on the network within 2days, and sports headphones were selected as the product. On the one hand, the participants were young college students, who would exhibit a certain level of demand for sports headphones, and they were familiar with them. On the other hand, the headphone components had a simple construction compared to other hardware products. The process of participation in innovation was to separate the whole product according to various functional requirements, and each function showed different preferences for the selection of the participants. The functional requirements were roughly divided into headphone size, tone quality processing, manipulation, memory, battery, wireless connection, feedback prompt, motion planning, etc. There was two-level selection for each function, such as the headphone size, which was divided into large, medium, and small. The size and shape of the earphones could be selected; these categories were divided into circle, ellipse, and thickness. There were 128 choices, each of which was published in graphic form, and brief descriptions of the advantages and disadvantages of each form were given. In addition, all options had a default manufacturer selection. In the selection process, there were two ways in which participants could interact with each other. First, the number of people choosing each option would be prompted once the participant had made the selection for that option. Second, a BBS forum was available for people to immediately discuss their choices. Afterward, the final product was developed and combined to complete the display.

#### Control Test

Social exclusion still used the form of self-reporting (Kim et al., 2013). There were three items using a 5-point scoring system. The reliability coefficient of the scale was 0.81, and there was a significant difference between the two groups (Mhigh=3.56, Mlow=2.61; *t*=5.72, *p*<0.05). This experiment also used the customer knowledge scale of Research 1 to construct a test (Wynder, 2007), and there was no significant difference between the two groups (Mhigh=4.09, Mlow=3.77; *t*=4.23, *p*=0.55).

#### Results

Customer-company identification was measured by the same scale used in Research 1. In this experiment, the participants were involved in the innovation process. To measure of customers’ willingness to participate in innovation, the number of participants who selected the default manufacturer option was converted into data. The more this option was chosen, the smaller was the customer’s willingness to participate in innovation, as customers’ willingness to participate in innovation is a process of psychological and physiological involvement (Bendapudi and Leone, 2003). When the participants selected the default manufacturer option, it can be interpreted as meaning that they did not want to devote more energy to understanding the process. Therefore, we can infer that their willingness to participate in innovation was not high.

**Figure 2:**
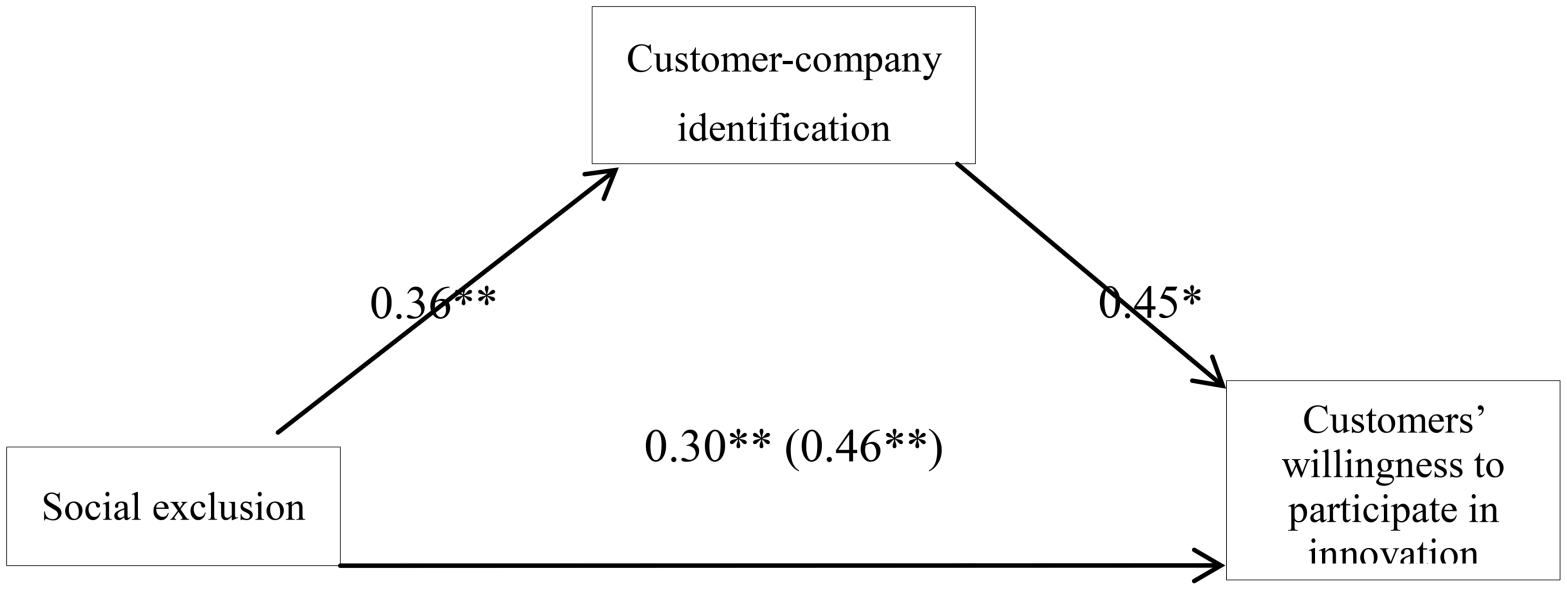
Customer-company identification mediation model and the regression coefficient of each path. ^*^p<0.05; ^**^p<0.01.

Social exclusion was treated as the independent variable, and the variance analysis was carried out for this variable along with customer-company identification and customers’ willingness to participate in innovation. The normalized regression coefficient of each path of the model is shown in Figure 2. The results showed that social exclusion had a significant impact on customer-company identification (*F*(1,87)=6.88, *p*<0.05) and customer’s willingness to participate in innovation (*F*(1,87)=5.09, *p*<0.05). Customer-company identification positively affected customers’ willingness to participate in innovation (*F*(1,87)=7.45, *p*<0.05). We use the same approach as in study 1 to test the intermediary role. The results showed that social exclusion had a significant positive predictive effect on customers’ willingness to participate in innovation (*β*=0.46, *p*<0. 05). When customer-company identification entered the regression equation, the regression coefficient of social exclusion for the customers’ willingness to participate in innovation declined, but it was still significant (*β*=0.30, *p*<0.05), indicating that customer-company identification played an intermediary role in the prediction of customers’ willingness to participate in innovation by social exclusion.

The experimental results support the hypotheses of this study. The correlation coefficient of social exclusion for the customers’ willingness to participate in innovation is higher in Research 2, and there are two possible reasons for this. One is the difference between the specific experiment process and the sample. The other is the situation in this study in which customer participation in innovation involves more interaction processes, better satisfies customers’ needs for social attribution, and also highlights the impact of customer-company identification on customers’ willingness to participate in innovation. In addition, the study used actual behavior data. The results indicate that the change is not significant. The main reason for this is that in an experimental situation, people do not need to devote considerable energy, and the difference between attitude and behavior is not highly significant. This is an issue that requires further attention in future research.

## Discussion

The experimental results support the hypotheses. Social exclusion affects the willingness of customers to participate in innovation, and customer-company identification plays an intermediary role in this relationship. From the perspective of social factors, the article conclusion partially explains the mechanism of customer participation in innovation. The first theoretical contribution of this paper is that it demonstrates social exclusion’s influence on customer participation in innovation in the context of the emerging social network and extends the antecedent research on customer participation in innovation to the social factor level (Ngo and O’cass, 2013). The second contribution is that it sheds light on the positive influence of a negative factor, such as social exclusion on customer participation in innovation, and expands the research perspective on customer participation. The third contribution is that it reveals the operational mechanism of the customer participation antecedents. Customer-company identification can play an intermediary role, which further supports the idea that the recognition is an important factor in customer participation (Bhattacharya and Sen, 2003). In terms of practical contributions, the findings of this study can inform enterprises who wish to carry out innovation management and participate in designing collaborative links between customers and enterprises to facilitate joint innovation. Customers can be important contributors to enterprise innovation (Brunswicker and Vanhaverbeke, 2015; Chen and Liu, 2020) but one challenge is how to motivate customers—who are not formally employed by the enterprise—to participate in innovation. One solution could be publicity to showcase an enterprise’s culture and products and further enhance customers’ enterprise identification, which could increase their willingness to participate in innovation. Social excluded customer will be more willing to participate in innovation. However, if enterprises adopt a strategy of rejecting customers, it is still a very risky behavior. For the sources of rejection, individuals will even adopt some excessive behavior, which is difficult to change to accept and produce a positive connection (DeWall et al., 2009b).

Although the experimental results of this study support the relevant hypotheses, some shortcomings of the research process should be noted. First, there are many ways to simulate social exclusion situations in psychological research (Kothgassner et al., 2014). The article uses two forms in the context of customer participation, and they pass the manipulation test. However, these situations still differ from actual market operations. The second shortcoming is the experimental process. With social exclusion, the intermediary role of customer-company identification is deduced, which has a strong theoretical basis. However, the customer-company identification that is described herein may also play a regulatory role, and there is no detailed distinction between these roles in this study. The third shortcoming is that the measurement of a customer’s willingness to participate in innovation in Research 2 is replaced by data that reflect the customer’s behavior. However, there are some differences between behavior and willingness, and some confusion factors may have existed between the two.

In future studies, the first objective would be to explore the influence of different sources of social exclusion (e.g., those of employees versus those of customers) and whether these have differential effects. Moreover, this article only focuses on the willingness of customers to participate in innovation, but from the enterprises’ perspective, the ability of customers to engage in innovation may have more practical value. Thus, future research is required to clarify the results of such innovation. Finally, the experimental approach in this study requires testing in other forms and situations to increase the external validity of the conclusions.

## Data Availability Statement

The raw data supporting the conclusions of this article will be made available by the authors, without undue reservation.

## Ethics Statement

The studies involving human participants were reviewed and approved by the School ethics committee. The patients/participants provided their written informed consent to participate in this study.

## Author Contributions

ZH is the leading author and prepares the manuscript and wrote the main part of the manuscript. MY helps in the data collection. ZC helps in the data analysis and methodology. LH conducted the literature review and wrote this part. GD proof-read the manuscript and helps to improve the quality of the manuscript. All authors contributed to the article and approved the submitted version.

## Funding

This research study was supported by the National Natural Science Foundation of China (grant no. 71672053) and National Social Science Foundation of China (grant no. 20BGL091).Research Center of Hubei Micro & Small Enterprises Development also provided corresponding funding.

## Conflict of Interest

The authors declare that the research was conducted in the absence of any commercial or financial relationships that could be construed as a potential conflict of interest.

## Publisher’s Note

All claims expressed in this article are solely those of the authors and do not necessarily represent those of their affiliated organizations, or those of the publisher, the editors and the reviewers. Any product that may be evaluated in this article, or claim that may be made by its manufacturer, is not guaranteed or endorsed by the publisher.
